# Internal cleavage and synergy with twisted gastrulation enhance BMP inhibition by BMPER

**DOI:** 10.1016/j.matbio.2018.08.006

**Published:** 2019-04

**Authors:** Michael P. Lockhart-Cairns, Karen Tzia Wei Lim, Alexandra Zuk, Alan R.F. Godwin, Stuart A. Cain, Gerhard Sengle, Clair Baldock

**Affiliations:** a Wellcome Centre for Cell-Matrix Research, Division of Cell-Matrix Biology and Regenerative Medicine, School of Biological Sciences, Faculty of Biology, Medicine and Health, University of Manchester, Manchester Academic Health Science Centre, UK; b Diamond Light Source, Harwell Science and Innovation Campus, Didcot, UK; c Center for Biochemistry, Medical Faculty, University of Cologne, Germany; d Center for Molecular Medicine, University of Cologne, Germany

## Abstract

Bone morphogenetic proteins (BMPs) are essential signalling molecules involved in developmental and pathological processes and are regulated in the matrix by secreted glycoproteins. One such regulator is BMP-binding endothelial cell precursor-derived regulator (BMPER) which can both inhibit and enhance BMP signalling in a context and concentration-dependent manner. Twisted gastrulation (Tsg) can also promote or ablate BMP activity but it is unclear whether Tsg and BMPER directly interact and thereby exert a synergistic function on BMP signalling. Here, we show that human BMPER binds to Tsg through the N-terminal BMP-binding region which alone more potently inhibits BMP-4 signalling than full-length BMPER. Additionally, BMPER and Tsg cooperatively inhibit BMP-4 signalling suggesting a synergistic function to dampen BMP activity. Furthermore, full-length BMPER is targeted to the plasma membrane via binding of its C-terminal region to cell surface heparan sulphate proteoglycans but the active cleavage fragment is diffusible. Small-angle X-ray scattering and electron microscopy show that BMPER has an elongated conformation allowing the N-terminal BMP-binding and C-terminal cell-interactive regions to be spatially separated. To gain insight into the regulation of BMPER bioavailability by internal cleavage, a disease-causing BMPER point mutation, P370L, previously identified in the acid-catalysed cleavage site, was introduced. The mutated protein was secreted but the mutation prevented intracellular cleavage resulting in a lack of bioactive cleavage fragment. Furthermore, mutant BMPER was extracellularly cleaved at a downstream site presumably becoming available due to the mutation. This susceptibility to extracellular proteases and loss of bioactive N-terminal cleavage fragment may result in loss of BMPER function in disease.

## Highlights

• BMPER and Tsg work in concert to increase the inhibition of BMP4 signalling.
• The N-terminal BMPER cleavage product is a more potent inhibitor of BMP4 activity.
• BMPER is elongated and flexible with distinct BMP- and HS-binding regions.
• A disease causing BMPER point mutation prevents internal auto-catalytic cleavage.

## Introduction

Bone morphogenetic proteins (BMPs) are members of the transforming growth factor beta (TGF-β) superfamily of secreted growth factors which are known to be key regulators of cellular behaviour [1]. The activity of BMPs is tightly controlled by extracellular modulators that promote or antagonize BMP activity. BMP-binding endothelial cell precursor-derived regulator (BMPER in human or Crossveinless-2 (CV2) in other species) and twisted gastrulation (Tsg) are distinct from other extracellular BMP regulators since they function as both agonists and antagonists of BMP activity. BMPER and Tsg play important roles at multiple developmental stages, such as patterning of the embryonic dorsal–ventral axis [2], or during skeletogenesis, cartilage and lung development [3,4]. In addition, BMPER is a key regulator of angiogenesis [5] as well as being required for vascular homeostasis [6].

**Fig. 1:**
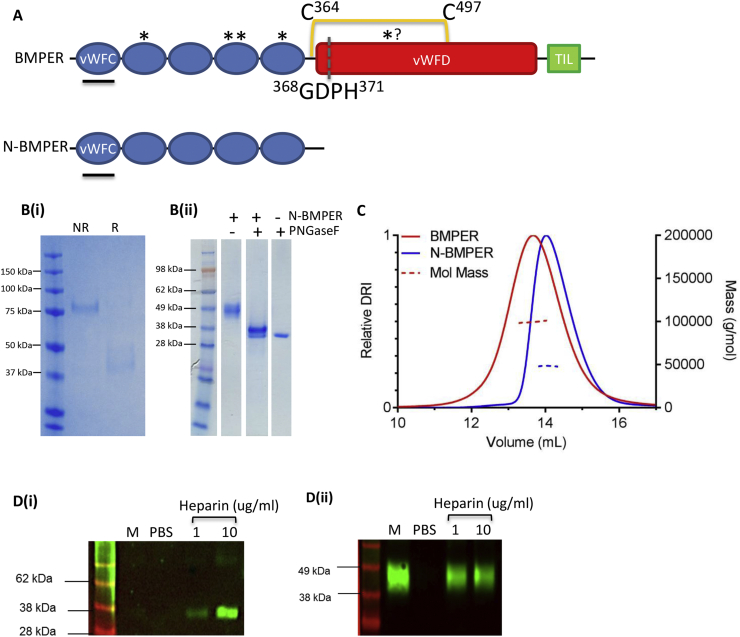
Recombinant expression and characterisation of BMPER proteins. (A) Schematic representation of the domain structures of full length (FL-) BMPER and C-terminally truncated N-BMPER (vWFC: von Willebrand Factor type C domain, vWFD: von Willebrand Factor type D domain, TIL: Trypsin inhibitor like domain). The BMP binding domain (vWFC1) is underlined in black, glycosylation sites are marked with asterisks (*) and the inter-domain disulphide bond between the vWFC5 and vWFD domains is marked in orange (C364-C497 [10]). The location of the GDPH acid-catalysed cleavage site is indicated by a grey dashed line. (B) Coomassie stained SDS-PAGE gels of purified (i) FL-BMPER (NR:non-reduced; R:reduced) under reducing conditions the protein dissociates due to internal autocatalytic cleavage but the cleavage fragments remain associated due to a intramolecular disulphide bond. (ii) N-BMPER before and after deglycosylation with PNGaseF under reducing conditions. (C) Multi-angle light scattering of FL-BMPER (red) and N-BMPER (blue). (D) Conditioned media (M) was collected from stable cell lines expressing (i) FL-BMPER and (ii) N-BMPER either with or without washing with PBS, 1 or 10 μg/mL of unfractionated heparin. The media was analysed by non-reduced western blotting probed with anti-His antibody.

BMPER mutations cause skeletal disorders such as Diaphanospondylodysostosis (DSD) [7], with phenotypic similarities to the BMPER-null mouse [8], and the milder Ischiospinal dysostosis [9]. One particular BMPER mutation, P370L, was identified at a site where acid-catalysed cleavage occurs [7]. While there is a general consensus that BMPER cleavage is important, the effect of cleavage and function of the cleavage products is controversial [[10], [11], [12]]. BMPER is composed of five von Willebrand factor type C (vWFC) domains, one von Willebrand factor type D (vWFD) domain and a Trypsin inhibitor like domain (TIL) domain (Fig. 1A). The conserved, acid-catalysed cleavage motif of BMPER, GDPH, is found within the vWFD domain where a disulphide bond straddling the cleavage site tethers the cleavage products together. The transport of BMPER through the extracellular matrix is hindered by its interaction with heparan sulphate proteoglycans (HSPGs) mediated by the vWFD domain, resulting in short range modulation of BMP signalling at the cell surface [10].

BMPER inhibits BMP activity by directly binding the growth factor via the vWFC1 domain which blocks the type I and II BMP receptor binding sites [13,14]. The pro-BMP activity of BMPER is mediated by displacing the BMP growth factor from the chordin-Tsg-BMP inhibitory complex. Thereby BMPER and chordin directly interact via the BMPER vWFC-1 and -4 domains and vWFC2 of chordin [11,15,16]. Although it is known that Tsg and chordin interact with high-affinity via domains vWFC2-3 of chordin [17], there is conflicting literature regarding a BMPER-Tsg interaction. A direct interaction was not observed between mouse Tsg and zebrafish BMPER when assayed by SPR [18]. However, mouse BMPER and Tsg were shown to interact by immunoprecipitation [15], which may potentially be mediated via an indirect interaction. Both antagonism and synergy between BMPER and Tsg have been reported previously [19,20] so it is still not clear how these modulators of BMP activity function in concert.

Therefore, in order to investigate whether a direct interaction between human BMPER and Tsg occurs in the modulation of BMP activity, we combined structural, biophysical and cell based approaches. We show that BMPER binds to Tsg with high affinity via the vWFC domains and that BMPER and Tsg work in concert to inhibit BMP4 activity. Furthermore, a recombinant BMPER fragment representing the N-terminal region served as a more effective BMP4 inhibitor than full-length BMPER. Interestingly, introducing the DSD-causing mutation P370L prevented acid-catalysed internal cleavage and rendered the secreted protein susceptible to extracellular cleavage but did not alter the bioactivity of the full-length protein. In addition, our structural and biophysical characterisation of BMPER revealed an elongated, flexible molecule.

## Results

### Purification of human BMPER, a modular glycoprotein

Human BMPER and the N-terminal five vWFC domains (N-BMPER) were expressed in mammalian cells and purified (Fig. 1A and B). Molecular mass estimates from multiangle light scattering (MALS) for full-length (FL-) BMPER and N-BMPER were 100 kDa and 48 kDa, respectively (Fig. 1C) which are larger than the predicted masses from sequence (72.5 kDa for FL- BMPER and 36.5 kDa for N-BMPER). However, this discrepancy may be explained by the presence of five predicted N-glycosylation sites in BMPER, four in the vWFC domains and one in the vWFD domain, adding additional mass upon glycosylation. Interestingly, a similar N-glycosylation site in vWFD is not utilised in chick CV2 [21]. Indeed, PNGase F digestion of N-BMPER resulted in a shift in mobility on SDS-PAGE to the expected size of ~36 kDa (Fig. 1B).

### The C-terminal region of BMPER mediates interaction with the cell surface

The C-terminal region of BMPER has been implicated in interactions with glycosaminoglycans (GAGs) such as HS. Zebrafish CV2 has been shown to bind to the cell surface via HSPGs [11], but this has not been investigated for human BMPER. BMPER expressing cells were dosed with unfractionated heparin, a more sulphated form of HS, to compete for BMPER interactions with the cell surface. When cells were incubated with increasing amounts of heparin there was an increase in the amount of FL-BMPER and the C-terminal region of BMPER in the media (Fig. 1Di). However, high levels of N-BMPER were secreted into the media and there was no increase in the amount of N-BMPER upon heparin addition (Fig. 1Dii). This indicates that N-BMPER does not interact with HS at the cell surface and that HS binding is mediated by C-terminal BMPER domains.

### BMPER binds to Tsg with high affinity via its N-terminal vWFC domains

**Fig. 2:**
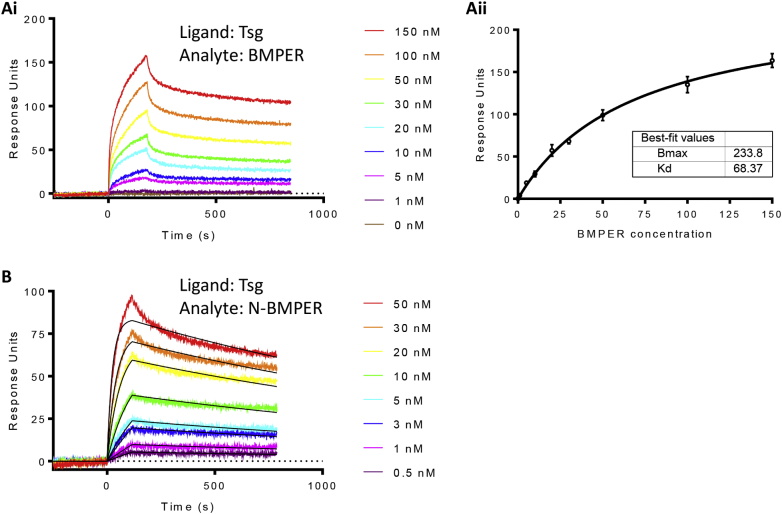
SPR analysis of BMPER binding to Tsg. Tsg was immobilised to the sensor chip, using amine coupling and concentrations of FL-BMPER (0–150 nM) and N-BMPER (0–50 nM) were flowed over as analytes. Sensorgrams showing real-time binding of FL-BMPER to Tsg (Ai) were subjected to equilibrium analysis (Aii). (B) N-BMPER interacts with Tsg according to a 1:1 Langmuir binding model (curve fits are overlaid in black). All experiments were performed in triplicate, representative data shown.

It has been previously shown that zebrafish CV2 binds to chordin with high affinity and thereby enhances BMP activity [16]. However, chordin is found in a ternary complex with Tsg and BMP; whether CV2/BMPER and Tsg also interact is unclear. Mouse Tsg and zebrafish CV2 did not bind to each other when assayed using SPR [18], however, mouse CV2 and Tsg were shown to interact by immunoprecipitation [15]. Therefore, in order to determine whether human BMPER and Tsg interact, surface plasmon resonance (SPR) interaction assays were employed. Indeed, FL-BMPER bound to immobilised Tsg with relatively high affinity (K_D_ 68.37 nM) (Fig. 2A, Table 1). In order to determine whether the vWFC domains, which are known to mediate the chordin-Tsg interaction, are required for this interaction, N-BMPER was subjected to SPR binding assays with immobilised Tsg. The obtained sensorgrams indicated that N-BMPER and Tsg interacted with very high affinity (K_D_ 0.55 nM) (Fig. 2B). The K_D_ values of FL-BMPER and N-BMPER binding to Tsg vary by two orders of magnitude, due to a slower on-rate (k_on_) of FL-BMPER, while the off rates (k_off_) for both BMPER variants were comparable (Table 1; Supplementary Fig. 1). The slower on-rate of FL-BMPER was probably caused by secondary BMPER-BMPER interactions on the SPR chip likely mediated by the vWFD domain which is present in FL-BMPER but not N-BMPER. The known interaction between chordin (vWFC2-3) and N-BMPER was also analysed confirming a high-affinity interaction between these regions (Table 1; Supplementary Fig. 2).

**Table 1:** Surface plasmon resonance data for interactions between Tsg or chordin (immobilised) and BMPER constructs (solution phase).

Ligand	Analyte	K_off_ (1/s)	K_on_ (1/M*s)	K_D_ (nM)	K_D_ obtained by
Tsg	BMPER	2.03 × 10^−4^	2.97 × 10^3^	68.37	Equilibrium analysis
Tsg	N-BMPER	2.28 × 10^−4^	4.15 × 10^5^	0.55	Langmuir 1:1 model
Chordin vWFC2-3	N-BMPER	8.22 × 10^−5^	4.04 × 10^5^	0.20	Langmuir 1:1 model

### BMPER and Tsg work in concert to inhibit BMP signalling

**Fig. 3:**
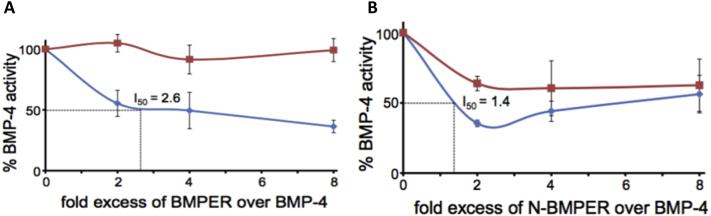
BMP bioactivity measurements in presence of Tsg and BMPER. Inhibition of BMP4 activity by addition of increasing amounts of BMPER (A) and N-BMPER (B) in presence or absence of 28 times molar excess of Tsg was monitored by alkaline phosphatase (ALP) production in C2C12 cells. All cells were treated with 1 nM of BMP4. Red squares: absence of Tsg; Blue diamonds: presence of Tsg. Error bars are from the standard deviation of experiments performed in triplicate.

BMPER can act as both an agonist and antagonist of BMP signalling in a context and concentration dependent manner [12]. Tsg also exerts promoting and inhibiting effects on BMP activity. Thereby, Tsg strengthens BMP inhibition by chordin or enhances chordin cleavage by tolloids [22]. To determine whether Tsg and BMPER are able to work in concert to inhibit or enhance BMP activity, we conducted inhibition assays using BMP4. In these assays, the formation of alkaline phosphatase (ALP) by C2C12 cells was measured upon administration of BMP4 plus additional increasing concentrations of FL-BMPER or N-BMPER, in the presence or absence of a molar excess of Tsg (Fig. 3). Tsg increased the inhibitory action of both FL-BMPER and N-BMPER suggesting that Tsg and BMPER act synergistically to inhibit BMP4 signalling. Interestingly, at the tested concentrations N-BMPER was a better BMP4 inhibitor than FL-BMPER suggesting that the C-terminal region attenuates BMP inhibition.

### Mutation of BMPER alters the internal cleavage site

**Fig. 4:**
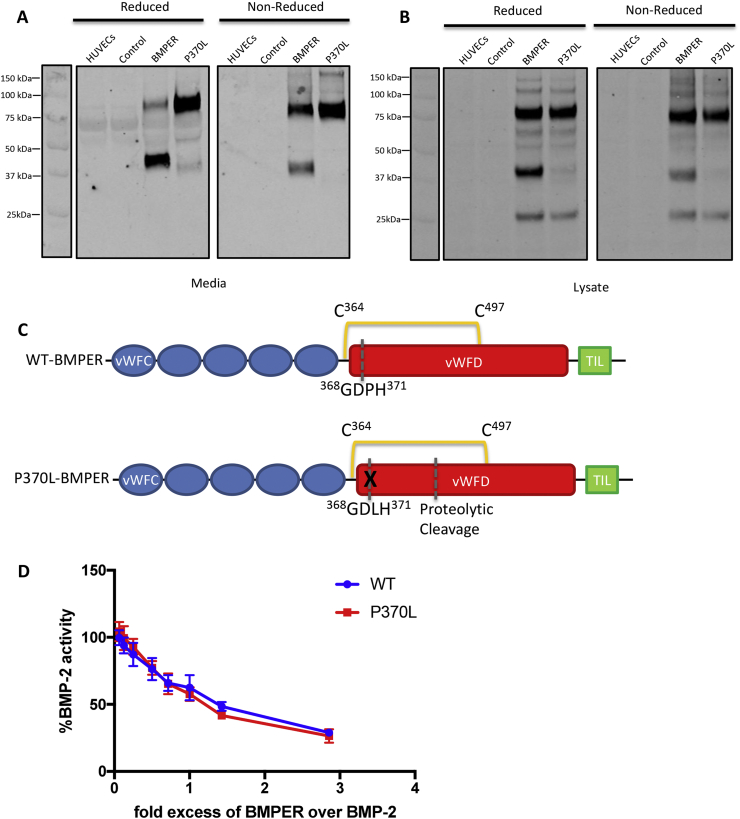
Secretion, activity and extracellular cleavage of BMPER-P370L. The conditioned media and cell lysates of BMPER and BMPER-P370L cell-lines and empty vector control cells were probed by Western blot using an anti-V5 antibody. Reduced and non-reduced conditioned media (A) and cell lysates (B) from transfected HUVECs. (C) Schematic diagram of BMPER structure indicating location of GDPH cleavage site and putative proteolytic cleavage site of P370L mutant. (D) Inhibition of BMP2 activity by addition of increasing amounts of purified FL-BMPER (blue) and BMPER-P370L (red) using a nanoLuciferase BRE-reporter in ATDC5 cells. All cells were treated with 10 nM of BMP2. Error bars are from the standard deviation of experiments performed in triplicate.

BMPER is secreted as both a full-length protein and a cleavage product upon utilisation of the G^368^DPH^371^ site, as shown in Fig. 1A. To investigate the role of BMPER cleavage, human umbilical vein endothelial cells (HUVECs) were stably transfected with lentivirus expressing BMPER and BMPER-P370L, a DSD-causing mutation which alters the GDPH site to GDLH. The similarity of the DSD phenotype to the BMPER null mouse suggests that the mutation results in loss of BMPER function. However, BMPER-P370L was secreted into the media with similar molecular weight to the full-length wildtype protein (~75 kDa) (Fig. 4A). Wildtype BMPER undergoes internal cleavage at the GDPH site; this cleavage product is apparent after the internal disulphide bond is reduced and can be observed at ~40 kDa. After reduction, BMPER-P370L still predominantly migrated at ~75 kDa indicating that cleavage at the GDPH site had not occurred albeit that a faint smaller molecular weight band was apparent (~35 kDa) (Fig. 4A). As expected, the acid-catalysed cleavage at the GDPH site occurred intracellularly for wildtype BMPER but not BMPER-P370L (Fig. 4B). Moreover, the ~35 kDa BMPER-P370L cleavage product was not present in the cell lysate so this cleavage must occur extracellularly. For both wildtype and mutant BMPER, a lower molecular weight species of ~25 kDa was observed in the cell lysate, suggestive of cleaved protein retained or degraded intracellularly (Fig. 4B). These data suggest that the BMPER-P370L is secreted, but the mutation prevents intracellular cleavage at the GDPH site (Fig. 4C).

To determine whether the P370L mutation alters the BMP-inhibitory capacity of FL-BMPER, we conducted inhibition assays using BMP2. For these assays, a nanoLuciferase reporter fused to a BMP-responsive element was stably transfected into human chondrogenic ATDC5 cell line. BMP2 plus increasing concentrations of FL-BMPER or BMPER-P370L were added to the ATDC5 cells and the luciferase signal measured (Fig. 4D). Both wildtype and P370L FL-BMPER inhibited BMP2 to the same level. These data suggest that internal BMPER cleavage is not required for BMP-inhibition by the full-length proteins but this does not take into account the secreted N-terminal cleavage fragment that is a more potent BMP inhibitor, which is produced with wildtype BMPER but not BMPER-P370L (Fig. 4A).

A small proportion of mutant BMPER is subsequently cleaved at a downstream site, most likely by an extracellular proteinase. The new cleavage site is located between the internal disulphide bond that tethers the wildtype GDPH cleavage products (Cys364-Cys497), as the ~35 kDa fragment is only observed after reduction (Fig. 4A). There is a putative furin/PACE recognition site, R^410^RTR^413^, which is a potential candidate for an extracellular cleavage site. To investigate whether the extracellular cleavage seen for BMPER-P370L is mediated by furin/PACE proteinases, residue R410A was mutated resulting in A^410^RTR^413^ ablating the furin site. Wildtype, P370L and double mutant P370LR410A were stably transduced into HEK293-EBNA cells. Mutation of the putative furin cleavage site did not change the pattern of intracellular or extracellular cleavage of mutant BMPER indicating that extracellular cleavage is not mediated by furin/PACE proteases (Supplementary Fig. 3).

### BMPER has an elongated structure separating the N- and C-terminal regions

**Fig. 5:**
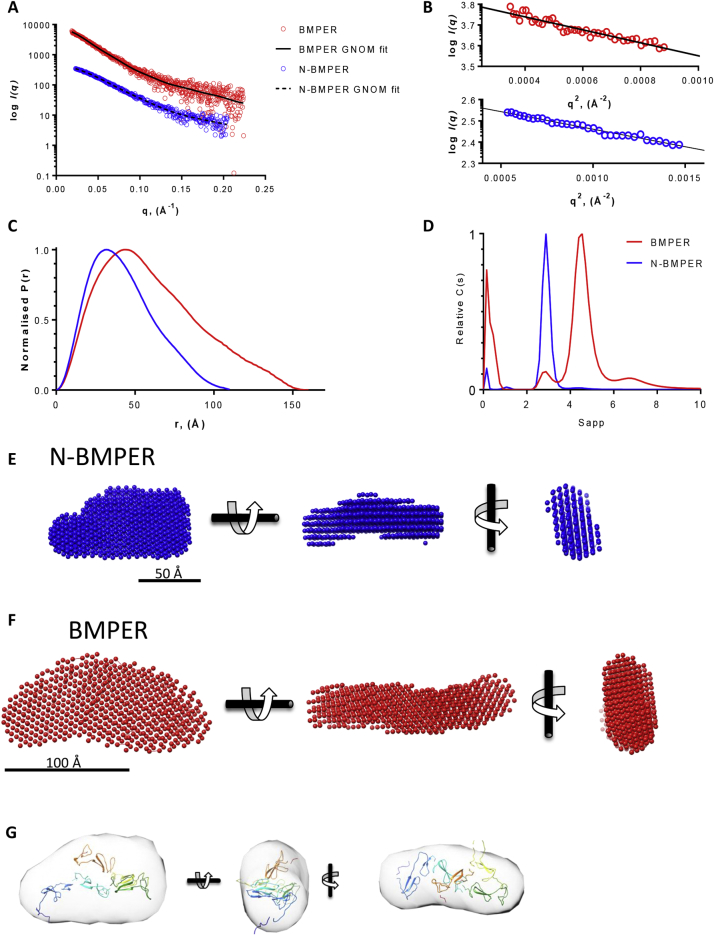
X-ray scattering and analytical ultracentrifugation of BMPER. (A) X-ray scattering data of N-BMPER (blue) and FL-BMPER (red) plotted with the indirect Fourier Transform fit. (B) The low q scattering data represented as a Guinier plot for N-BMPER (blue) and FL-BMPER (red). (C) The pair-distance distribution functions P(r) for N-BMPER (blue) and FL-BMPER (red). (D) Sedimentation velocity analytical ultracentrifugation hydrodynamic analysis for N-BMPER (blue) and FL-BMPER (red). Using DAMMIF, ab initio models were generated for N-BMPER and BMPER (E and F respectively) with scale bars of 50 and 100 Å, respectively. (G) Rigid body modelling of the five vWFC domains in N-BMPER with Coral.

To determine the relative arrangements of the HSPG-binding C-terminal region and BMP-binding N-terminal region of BMPER, the structure of BMPER was analysed by small angle X-ray scattering (SAXS) (Fig. 5). The radius of gyration (R_g_) and the maximal dimension (Dmax) were 48.0 Å and 160 Å for BMPER and 33.2 Å and 111 Å for N-BMPER (Fig. 5B,C; Table 2). The profiles of the P(r) plots show elongated structures for both constructs which is confirmed in the normalised Kratky plot (Fig. 5C; Supplementary Fig. 4). Analysis of flexibility plots suggests a degree of flexibility most likely for the linkers between domains (Supplementary Fig. 4). Analytical ultracentrifugation (AUC) data confirmed that BMPER is an elongated protein and the frictional ratio (*f*/*f*_0_) shows that BMPER and N-BMPER have the same level of elongation (1.73 versus 1.74) (Fig. 5D; Table 2). The AUC data for FL-BMPER also shows small amounts of low molecular weight and higher order species. These are most likely the C-terminal BMPER cleavage product that has co-purified with FL-BMPER and BMPER oligomers. In contrast N-BMPER was a single species. Ab initio models for BMPER and N-BMPER were constructed from the SAXS data using DAMMIF [23] (Fig. 5 E, F; Supplementary Fig. 4). Generating homology models for each of the vWFC domains of N-BMPER enabled rigid body modelling of N-BMPER using CORAL [24]. Ten models were generated and a representative model was selected based on best fit to the N-BMPER data (Fig. 5G).

**Table 2:** Measured hydrodynamic and structural parameters from MALS, AUC and SAXS compared with calculated hydrodynamic and structural parameters from the EM 3D reconstruction and SAXS models.

		BMPER	N-BMPER
*Measured with:*			
MALS	R_h_ (Å)	51	40
	MW (kDa)	100.4	48.3
AUC	R_h_ (Å)	53	41
	f/f_0_	1.73	1.74
	S_w,20_	4.59	2.92
SAXS	R_g_ (Å)	48.0	33.2
	D_max_ (Å)	160	111
	Volume (Å^3^)	320,000	140,000
	Model resolution (Å)	44 (± 3)	35 (± 3)

*Calculated from:*			
EM	R_h_ (Å)	54.79	N/A
	S_w,20_	4.09	N/A
	R_g_ (Å)	49.39	N/A
	D_max_ (Å)	166.3	N/A
SAXS	R_h_ (Å)	48.8	36.5
	f/f_0_	1.62	1.54
	S_w,20_	4.89	3.09

**Fig. 6:**
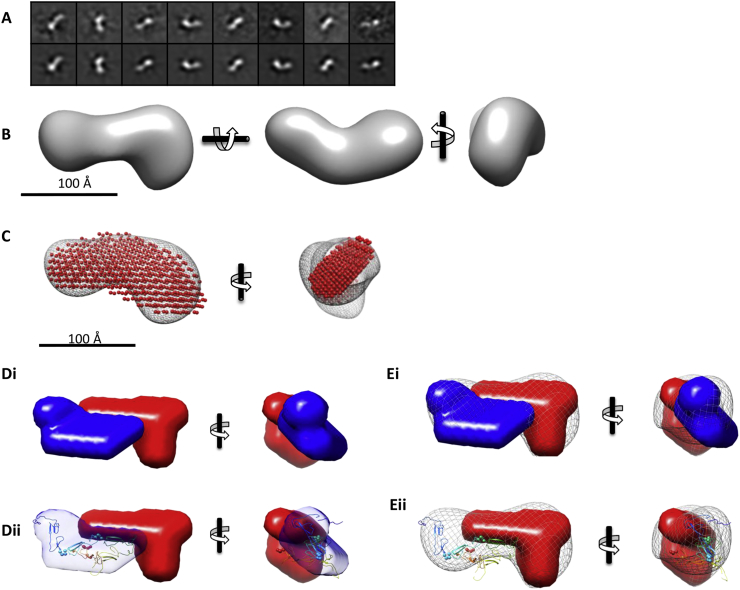
Electron Microscopy and BMPER domain modelling. Negatively stained electron microscopy images were recorded of FL-BMPER. (A) Representative class averages of FL-BMPER (top) with corresponding re-projections representing different Euler angles (bottom). Box size = 25.8 × 25.8 nm. (B) 3D reconstruction of BMPER from the EM data. (C) The SAXS bead model of BMPER from Fig. 5 was fitted into the EM electron density using UCSF Chimera. (Di) Multi-phase modelling of the N- and C-terminal regions of BMPER was performed using Monsa with the FL-BMPER and N-BMPER SAXS data. N-BMPER is represented by blue density and the C-terminal BMPER region is shown as red density and (Dii) with the rigid body model of vWFC domains 1–5 fitted into the N-BMPER density. (E) The Monsa model shown in (D) is superimposed into the electron density for FL-BMPER (i) with the rigid body model of N-BMPER included in place of density (ii).

Negative stain EM was also used to investigate the structure of BMPER. Particles were picked and classified allowing 3D model reconstruction from 3000 particles (Supplemental Fig. 5A, B). This initial model was refined iteratively to generate a final model with model projections similar to the corresponding classes (Fig. 6A and B). The 3D reconstruction was elongated with a bulge at one end which showed overall structural agreement to the ab initio SAXS model (Fig. 6C). Given that SAXS data were collected for both BMPER and N-BMPER it was possible to generate a multi-phase ab initio model using MONSA [25] from the contributions of each volume which provided a model of N-BMPER localisation within the whole BMPER structure (Fig. 6D). The N-BMPER rigid body model (Fig. 5G) aligned well to the N-BMPER density in the multi-phase ab initio model (Fig. 6Dii). The multi-phase ab initio model was compared with the EM structure which showed a good overall fit between the BMPER models generated from these different techniques (Fig. 6E). To compare hydrodynamic and structural data, hydrodynamic parameters were calculated in silico from the EM map using Hydromic [26], em2dam and Crysol [27] and from the SAXS bead models using SOMO [28,29]. All parameters were consistent from these methods providing further confidence in the structure (Table 2).

## Discussion

In this study we aimed to gain further insight into the molecular requirements for BMPER as modulator of BMP activity. Human FL- and N-BMPER are modular glycoproteins which were expressed for these studies. The N-terminal vWFC domains are glycosylated whereas the C-terminal region binds to HSPGs at the cell surface. The purification yield of FL-BMPER was increased dramatically when heparin was added to cells before harvesting conditioned media as FL-BMPER was competed off the cell surface. This approach may increase the yield of other recombinantly expressed extracellular matrix proteins which interact with the cell surface. The crystal structure of the vWC1 domain of CV2 in complex with BMP2 has been determined which demonstrates how BMP2 binds to this domain [14] but the structure does not provide details of the conformation of full-length BMPER. Our SAXS and EM data show that both N-BMPER and FL-BMPER are equally elongated and are flexible in solution. These data are consistent with an extended flexible modular glycoprotein where the N-terminal BMP/Tsg binding region is spatially separated from the C-terminal cell binding region for functionality.

The binding between Tsg and BMPER was investigated as there are conflicting reports in the literature regarding whether a direct interaction occurs between these two molecules. For instance Zhang and co-workers did not observe binding of zebrafish CV2 or any of the individual vWFC domains to mouse Tsg [18] but other groups did show that mouse Tsg interacted with mouse CV2 in immunoprecipitation experiments [15] although this could be mediated by an indirect interaction. However, we found using SPR that BMPER binds directly to Tsg with high affinity and this interaction is mediated by the N-terminal vWFC domains. The K_D_ of the N-BMPER/Tsg interaction is lower than that for FL-BMPER/Tsg which initially suggested a higher affinity interaction, but the off-rates for the two interactions are the same suggesting that the on-rate for the N-BMPER/Tsg interaction is faster. Furthermore, there is a secondary weak interaction seen for FL-BMPER/Tsg association which gives rise to a biphasic association curve. This is consistent with a secondary binding event between FL-BMPER/FL-BMPER after the initial interaction between FL-BMPER/Tsg. Indeed AUC shows the presence of FL-BMPER oligomers suggesting the full-length protein has a greater potential for self-association than N-BMPER. Together these data suggest that the interaction between Tsg and N-BMPER or FL-BMPER has the same affinity but FL-BMPER can self-associate once bound to Tsg. As N-BMPER does not bind to itself in the same manner this self-association is likely to be mediated by the vWFD or TIL domains.

Our BMP inhibition data show that the antagonistic action of BMPER is increased in the presence of Tsg. We have already shown that Tsg can inhibit BMP4 signalling directly by ~20% [17] whereas FL-BMPER alone is not a very effective inhibitor of BMP4 signalling in these cell-based assays (Fig. 3A). However, in conjunction, FL-BMPER and Tsg showed a greater inhibition of BMP4 signalling (~50%; I_50_ 2.6) (Fig. 3) indicating that they work in concert to inhibit BMP4 signalling. These data are consistent with observations in Xenopus where the anti-BMP activity of CV2 increased with co-injection of Tsg [15] and for mouse fibroblasts where CV2 and Tsg acted together to inhibit BMP4 signalling [30].

We found that N-BMPER was a more potent inhibitor of BMP signalling than FL-BMPER and also that N-BMPER was secreted and not tethered to the cell surface which could contribute to its increased inhibitory action. The vWFD of BMPER has been implicated in interactions with HSPGs and therefore the full-length protein would be less able to diffuse away from the cell surface which could result in increased BMP inhibition as HS has been implicated in the regulation of growth factor binding and signalling in the matrix and at the cell surface [31]. These data are in contrast to findings in *Drosophila* where a N-CV2 construct was only weakly active in crossvein formation in the wing [10]. Furthermore, *drosophila* N-CV2 did not interact with dpp by immunoprecipitation but zebrafish CV2 vWFC domains do bind to BMPs [11,18] suggesting that there are species differences.

Other proteins with an acid catalysed ‘GDPH’ cleavage motif, such as Repulsive guidance molecules, are not secreted when the motif is disrupted, presumably as it is required for the vWFD domain to fold correctly [32]. However, BMPER-P370L is secreted but cleaved extracellularly which we assume is by proteolytic activity. The cleavage site appears to be down-stream of the GDPH motif but the small decrease in mass would suggest that cleavage still occurs in the vWFD domain. A potential furin/PACE cleavage site, R^410^RTR^413^, was identified as a potential candidate for extracellular cleavage but mutation of the furin recognition sequence did not prevent extracellular cleavage of mutant BMPER indicating that cleavage is not mediated by Furin/PACE proteases. The new cleavage site is between the internal disulphide bond that tethers the wildtype GDPH cleavage products (Cys364-Cys497), as the fragment is only observed after reduction. As the mutant protein is more susceptible to proteolysis, the sequence between the disulphide bond was analysed which indicated potential elastase-2 and MMP9 sites [33]. Potentially the P370L mutation has perturbed the fold of the vWFD domain to expose a cryptic cleavage site that would otherwise be hidden within the domain structure. Increased susceptibility to extracellular proteases could result in degradation of the mutant BMPER protein. Furthermore, the mutant protein may also have an increased propensity for aggregation as a higher molecular weight species is observed on SDS-PAGE (Fig. 4A).

**Fig. 7:**
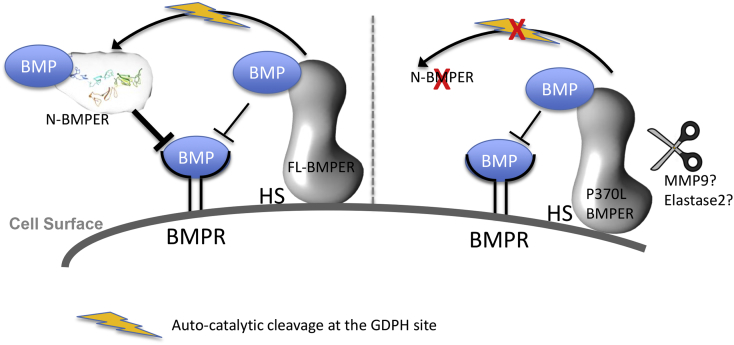
Model of BMPER mechanism and effect of P370L mutation. FL-BMPER is bound at the cell surface by HSPGs/glypicans. Auto-catalytic cleavage occurs, most BMPER remains intact due to the internal disulphide bond but some is liberated. The N-terminal cleavage fragment, N-BMPER, is a soluble, secreted protein, not bound to the cell surface and is a better inhibitor of BMP4, perhaps due to its increased solubility/diffusibility. The P370L mutation prevents auto-catalytic cleavage but the mutant protein is still secreted and inhibits BMP with the same efficacy as wildtype FL-BMPER. Some mutant protein is cleaved extracellularly at a downstream site. The cleavage site is before Cys497 and cleavage is not mediated by furin/PACE proteases. The mutant protein appears less stable, i.e. more susceptible to proteolytic cleavage and aggregation, and the ablation of the auto-catalytic cleavage site results in loss of the N-BMPER inhibitory fragment.

Together our data show that after auto-catalytic cleavage of FL-BMPER, most BMPER remains intact due to the internal disulphide bond but some is liberated (Fig. 7). The N-terminal cleavage fragment, N-BMPER, is soluble, not bound to the cell surface and is a better inhibitor of BMP4. The P370L mutation prevents auto-catalytic cleavage but the full-length mutant protein is still secreted and inhibits BMP with the same efficacy as wildtype BMPER. However, the mutant protein is less stable, i.e. more susceptible to proteolytic cleavage and aggregation, and there is less N-BMPER inhibitory fragment due to ablation of the auto-catalytic cleavage site, together resulting in reduced BMPER function in disease.

## Materials & methods

### Cloning, expression and purification of recombinant proteins

Full-length BMPER and N-BMPER, based on NCBI Sequence: NM_133468.4, were synthesised by Invitrogen GeneArt Gene Synthesis. Both constructs had a C-terminal, thrombin cleavable 6-His tag and were cloned into the pCEP-Pu/AC7 vector and transfected into HEK293 EBNA cells as described previously [34]. The P370L and R410A mutations were introduced into FL-BMPER using site-directed mutagenesis. For secretion studies, the native signal sequence of BMPER was added through overlap extension PCR using GeneArt String DNA fragments. The constructs were cloned into the pCDH vector with C-terminal V5-His tag for co-transfection with lentiviral packaging vectors pMD2.G and psPAX2. Virus containing media was used to transduce either HEK293-EBNA or HUVEC cells (Cellworks). Transduced cells were then sorted by FACS to obtain BMPER and BMPER-P370L expressing cells. Tsg and chordin were expressed and purified as described previously [17,34]. Protein was harvested from serum free media and purified by nickel affinity chromatography followed by size exclusion chromatography (SEC) in 10 mM Tris HCl pH 7.4, 500 mM NaCl. Where needed samples were concentrated using Vivaspin centrifugal concentrators (Sartorius). N-BMPER was deglycosylated using PNGaseF (NEB) in denaturing conditions.

### BMP inhibition assays

For ALP assays, C2C12 cells were seeded into 96-well plates at a density of 30,000 cells per well. BMP stimulation was carried out in triplicate with 1 nM BMP4 (R&D Systems) in the presence or absence of BMPER, N-BMPER, both BMPER and Tsg and both N-BMPER and Tsg for 72 h. Tsg was maintained at a 28-fold molar excess to BMP4 to achieve a response [17] while BMPER variants were titrated from 0 to 8-fold molar excess. After stimulation, cell layers were lysed and 100 μL alkaline phosphatase yellow (*p*-nitrophenyl phosphate) liquid substrate (Sigma-Aldrich) was added to 50 μL of each lysate and incubated for 20 min at room temperature followed by measurement of activity in a TECAN infinite M1000 reader (Dynamic Instruments) at 405 nm.

A chondrogenic cell line (ATDC5) containing a BMP reporter was generated using an established lentiviral method (SBI Systems Bioscience). The bone morphogenic response element (BRE) [35] was cloned upstream of a NanoLuc Luciferase (Promega) P2A-tagRFP sequence resulting in the dual luminescent/fluorescent reporter. Lentiviral particles were generated using HEK293T cells as described previously [36]. ATDC5 cells were infected with virus and selected by fluorescence-activated cell sorting (FACSAria Fusion, Beckon-Dickenson). Cells were seeded into 96-well plates at a density of 30,000 cells per well. BMP stimulation was carried out in triplicates with 10 nM BMP2 (R&D Systems) in the presence or absence of BMPER and BMPER-P370L for 72 h. After stimulation, cell layers were lysed and Nano-Glo® Luciferase reagent (Promega) added and the luciferase signal detected using a FL_x_ 800 Microplate Fluorescence Reader (BioTex Instruments Inc).

### Heparin titration assay

To an 80% confluent 6 well plate of BMPER or N-BMPER expressing cells, heparin (Iduron) was added at 1 μg/mL or 10 μg/mL, or washed with PBS as a control. The cells were incubated for 48 h before the media was analysed by Western Blot.

### Western blot antibodies

BMPER and N-BMPER constructs in pCEP-Pu/AC7 transfected cells were detected using a His Tag antibody (1:10,000, R & D Systems), whilst BMPER, BMPER-P370L, BMPER P370LR410A constructs in pCDH transduced cells were detected using a V5 tag antibody (1:5000, Bio-Rad).

### Multi-angle light scattering (MALS)

Samples were loaded onto a Superdex 200 10/300 GL column, running at a flow rate of 0.75 mL/min, equilibrated with 10 mM Tris HCl pH 7.4, 500 mM NaCl. Eluted samples passed through a DAWN Wyatt Helios II 18-angle laser photometer with one of the detectors replaced with a Wyatt QELS detector coupled to a Wyatt Optilab rEX refractive index detector. The molecular mass and hydrodynamic radii of the resulting peaks were analysed using Astra 6.1 (Wyatt, Santa Barbara, USA).

### Analytical ultracentrifugation (AUC)

Sedimentation velocity AUC of BMPER and N-BMPER was carried out in an XL-A Ultracentrifuge with an An50Ti-4-hole rotor (Beckman Coulter). Samples were measured at a wavelength of 230 nm at 45000 rpm at 20 °C where sedimentation was scanned every 90 s for 200 scans. Data was analysed with Sedfit [37] and Sednterp (http://sednterp.unh.edu/).

### Small-angle X-ray scattering (SAXS)

Samples of BMPER and N-BMPER were concentrated to 3 and 10 mg/mL, respectively. SAXS intensity data, *I(q)* versus *q* (*q* = 4*π*.  *sin* 2*θλ*), of BMPER were collected using SEC-SAXS on beamline B21 at Diamond Light Source and N-BMPER at beamline BM29 at the ESRF [38]. At B21, 50 μL of BMPER was loaded onto a Shodex KW-403 SEC column at 0.16 mL/min. SAXS data were collected at 1 s intervals on a Pilatus 2 M detector at a distance of 3.9 m and wavelength of 1 Å. At BM29, 50 μL of N-BMPER was loaded onto a Superdex 200 increase 3.2/300 SEC column at 0.075 mL/min. SAXS data were collected at 1 s intervals on a Pilatus 1 M detector at a distance of 2.9 m and wavelength of 0.992 Å. At both beamlines, the SEC eluent was flowed through the X-ray beam and the buffer used as the background was collected after one SEC column volume. For each beamline, data were reduced using in-house software. Subtractions of the SEC-SAXS data were completed for each frame across the elution peak and the radius of gyration (*R*_g_) and the integral of intensity ratio to background were plotted. The data were scaled, merged and averaged for each frame with a consistently similar *R*_g_. All further processing and analysis of data was carried out using ScÅtter (http://www.bioisis.net/scatter).

### SAXS data analysis and modelling

Bead models were generated for BMPER and N-BMPER using DAMMIF [23] in slow mode through ScÅtter. The calculated curves from bead models were compared to the experimental data and the agreement was shown by χ2 values ~1.126 and 0.213. The models from 23 independent DAMMIF runs were averaged using the DAMAVER suite with a mean normalised spatial discrepancy (NSD) of 0.672 ± 0.04 and 0.674 ± 0.06 (standard deviation) for BMPER and N-BMPER respectively. For multi-phase ab initio modelling, 20 biphasic models were generated using MONSA with an annealing schedule factor of 0.9 with a maximum of 500 annealing steps. Phase one was defined as N-BMPER and phase two defined by the volume difference between BMPER and N-BMPER, equating to the C-terminal contribution. The outputs from each run were separated into phases before averaging the models for each phase using adapted scripts from [39]. DAMMIF and MONSA bead models were visualized by generating an electron density map of 20 Å resolution in UCSF Chimera [40]. Homology models of individual vWFC domains (based on PDB accession code 3BK3 [14]) were generated with SWISSMODEL and CORAL [24] was run 10 times using five vWFC domains with resulting models fitting the data well. A single model was chosen to represent N-BMPER based on comparison of the theoretical R_g_ and D_max_ with the experimental data. The fit to the N-BMPER ab initio model was determined by ten global searches using UCSF Chimera [40].

### Electron microscopy (EM) and single particle analysis

BMPER (10 μg/mL) was adsorbed onto glow-discharged carbon-coated grids and stained with 4% (*w*/*v*) uranyl acetate (pH 4.7) and grids were observed using a FEI Tecnai12 Twin. Images were recorded at 120 keV under low dose conditions (<10 e−/Å2) on a 2048 × 2048 pixel Tietz TVIPS F214A camera (TVIPS) at 30,000 × magnification (2.8 Å/pixel) between −0.2 and − 2.0 μm defocus. Eman2 [41] was used for particle picking and image processing. Images were CTF corrected by phase flipping and the total number of particles in the dataset was 6000, picked by semi-automated picking. Particles were 2D classified by reference free alignment in 32 classes. Good classes were used to generate an initial model before five rounds of iterative refinement, using projection matching, was used to produce a self-consistent 3D reconstruction of BMPER. The resolution of the model was 35.8 Å, determined from the Fourier Shell Correlation (FSC) with a 0.143 cut off (Supplementary Fig. 5C).

### Calculated structural and hydrodynamic parameters of EM and SAXS models

The EM 3D reconstruction was converted to a bead model using EM2DAM from the ATSAS suite [42] at a threshold of 0.77. SAXS parameters from this model were calculated using Crysol [27]. Hydrodynamic parameters were calculated for the EM 3D reconstruction using Hydromic [26] at a threshold of 0.77 and a spacing of 2.8 Å/pixel. Hydrodynamic parameters of SAXS models were calculated with US-SOMO by SMI and ZENO methods [28,29].

### Surface plasmon resonance (SPR)

Protein-protein interactions were measured on a ProteOn XPR36 (Bio-Rad Laboratories) in 10 mM phosphate pH 7.4, 137 mM NaCl, 2.7 mM KCl and 0.005% Tween-20 at 25 °C. Tsg was immobilised on a GLC chip (Bio-Rad Laboratories) via amine coupling and blocked with ethanolamine. BMPER and N-BMPER were diluted in buffer to desired concentrations and injected onto the surface of the chip in increasing concentrations with a regeneration step of 2 M glycine pH 2.2 between each concentration. Kinetic data were calculated on the ProteOn Manager software (Bio-Rad Laboratories) fitting.

## Appendix A Supplementary data

Supplementary figures Image 1

## References

[1] Weiss A., Attisano L. (2013). The TGFbeta superfamily signaling pathway. Wiley Interdiscip. Rev. Dev. Biol..

[2] Langdon Y.G., Mullins M.C. (2011). Maternal and zygotic control of zebrafish dorsoventral axial patterning. Annu. Rev. Genet..

[3] Lorda-Diez C.I., Montero J.A., Rodriguez-Leon J., Garcia-Porrero J.A., Hurle J.M. (2013). Expression and functional study of extracellular BMP antagonists during the morphogenesis of the digits and their associated connective tissues. PLoS One.

[4] Zhou Y. (2018). Extracellular matrix in lung development, homeostasis and disease. Matrix Biol..

[5] Heinke J. (2008). BMPER is an endothelial cell regulator and controls bone morphogenetic protein-4-dependent angiogenesis. Circ. Res..

[6] Moser M., Patterson C. (2005). Bone morphogenetic proteins and vascular differentiation - BMPing up vasculogenesis. Thromb. Haemost..

[7] Funari V.A. (2010). BMPER mutation in diaphanospondylodysostosis identified by ancestral autozygosity mapping and targeted high-throughput sequencing. Am. J. Hum. Genet..

[8] Ikeya M. (2006). Essential pro-bmp roles of crossveinless 2 in mouse organogenesis. Development.

[9] Kuchinskaya E. (2016). Extending the phenotype of BMPER-related skeletal dysplasias to ischiospinal dysostosis. Orphanet. J. Rare Dis..

[10] Serpe M. (2008). The BMP-binding protein Crossveinless 2 is a short-range, concentration-dependent, biphasic modulator of BMP signaling in Drosophila. Dev. Cell.

[11] Rentzsch F., Zhang J., Kramer C., Sebald W., Hammerschmidt M. (2006). Crossveinless 2 is an essential positive feedback regulator of Bmp signaling during zebrafish gastrulation. Development.

[12] Kelley R. (2009). A concentration-dependent endocytic trap and sink mechanism converts Bmper from an activator to an inhibitor of Bmp signaling. J. Cell Biol..

[13] Fujisawa T., Huang Y., Sebald W., Zhang J.L. (2009). The binding of von Willebrand factor type C domains of Chordin family proteins to BMP-2 and Tsg is mediated by their SD1 subdomain. Biochem. Biophys. Res. Commun..

[14] Zhang J.L. (2008). Crystal structure analysis reveals how the Chordin family member crossveinless 2 blocks BMP-2 receptor binding. Dev. Cell.

[15] Ambrosio A.L. (2008). Crossveinless-2 is a BMP feedback inhibitor that binds Chordin/BMP to regulate Xenopus embryonic patterning. Dev. Cell.

[16] Zhang J.-L. (2010). Binding between Crossveinless-2 and Chordin Von Willebrand factor type C domains promotes BMP signaling by blocking chordin activity. PLoS One.

[17] Troilo H. (2016). Structural characterization of twisted gastrulation provides insights into opposing functions on the BMP signalling pathway. Matrix Biol..

[18] Zhang J.-L., Huang Y., Qiu L.-Y., Nickel J., Sebald W. (2007). von Willebrand factor type C domain-containing proteins regulate bone morphogenetic protein signaling through different recognition mechanisms. J. Biol. Chem..

[19] Heinke J. (2013). Antagonism and synergy between extracellular BMP modulators Tsg and BMPER balance blood vessel formation. J. Cell Sci..

[20] Ikeya M. (2010). Cv2, functioning as a pro-BMP factor via twisted gastrulation, is required for early development of nephron precursors. Dev. Biol..

[21] Kamimura M., Matsumoto K., Koshiba-Takeuchi K., Ogura T. (2004). Vertebrate crossveinless 2 is secreted and acts as an extracellular modulator of the BMP signaling cascade. Dev. Dyn..

[22] Larrain J. (2001). Proteolytic cleavage of Chordin as a switch for the dual activities of twisted gastrulation in BMP signaling. Development.

[23] Franke D., Svergun D.I. (2009). DAMMIF, a program for rapid ab-initio shape determination in small-angle scattering. J. Appl. Crystallogr..

[24] Petoukhov M.V. (2012). New developments in the ATSAS program package for small-angle scattering data analysis. J. Appl. Crystallogr..

[25] Svergun D. (1999). Restoring low resolution structure of biological macromolecules from solution scattering using simulated annealing. Biophys. J..

[26] Garcia De La Torre J., Llorca O., Carrascosa J.L., Valpuesta J.M. (2001). HYDROMIC: prediction of hydrodynamic properties of rigid macromolecular structures obtained from electron microscopy images. Eur. Biophys. J..

[27] Svergun D., Barberato C., Koch M.H.J. (1995). CRYSOL - a program to evaluate X-ray solution scattering of biological macromolecules from atomic coordinates. J. Appl. Crystallogr..

[28] Brookes E., Demeler B., Rocco M. (2010). Developments in the US-SOMO bead modeling suite: new features in the direct residue-to-bead method, improved grid routines, and influence of accessible surface area screening. Macromol. Biosci..

[29] Brookes E., Demeler B., Rosano C., Rocco M. (2010). The implementation of SOMO (SOlution MOdeller) in the UltraScan analytical ultracentrifugation data analysis suite: enhanced capabilities allow the reliable hydrodynamic modeling of virtually any kind of biomacromolecule. Eur. Biophys. J..

[30] Zakin L., Metzinger C.A., Chang E.Y., Coffinier C., De Robertis E.M. (2008). Development of the vertebral morphogenetic field in the mouse: interactions between Crossveinless-2 and twisted gastrulation. Dev. Biol..

[31] Patel V.N., Pineda D.L., Hoffman M.P. (2017). The function of heparan sulfate during branching morphogenesis. Matrix Biol..

[32] Bell C.H. (2013). Structure of the repulsive guidance molecule (RGM)-neogenin signaling hub. Science.

[33] Song J. (2012). PROSPER: an integrated feature-based tool for predicting protease substrate cleavage sites. PLoS One.

[34] Troilo H. (2014). Nanoscale structure of the BMP antagonist chordin supports cooperative BMP binding. Proc. Natl. Acad. Sci. U. S. A..

[35] Korchynskyi O., ten Dijke P. (2002). Identification and functional characterization of distinct critically important bone morphogenetic protein-specific response elements in the Id1 promoter. J. Biol. Chem..

[36] Cain S.A., Mularczyk E.J., Singh M., Massam-Wu T., Kielty C.M. (2016). ADAMTS-10 and -6 differentially regulate cell-cell junctions and focal adhesions. Sci. Rep..

[37] Schuck P. (2000). Size-distribution analysis of macromolecules by sedimentation velocity ultracentrifugation and lamm equation modeling. Biophys. J..

[38] Pernot P. (2013). Upgraded ESRF BM29 beamline for SAXS on macromolecules in solution. J. Synchrotron Radiat..

[39] Rambo R.P. (2015). Resolving individual components in protein-RNA complexes using small-angle X-ray scattering experiments. Methods Enzymol..

[40] Pettersen E.F. (2004). UCSF chimera - a visualization system for exploratory research and analysis. J. Comput. Chem..

[41] Tang G. (2007). EMAN2: an extensible image processing suite for electron microscopy. J. Struct. Biol..

[42] Franke D. (2017). ATSAS 2.8: a comprehensive data analysis suite for small-angle scattering from macromolecular solutions. J. Appl. Crystallogr..

