# Supplementary material for: Internal cleavage and synergy with twisted gastrulation enhance BMP inhibition by BMPER
Source: Matrix Biol. 2019 Apr;77:73–86. doi: 10.1016/j.matbio.2018.08.006 (PMC6456722; doi:10.1016/j.matbio.2018.08.006)
Supplement: Supplementary file 1 — Supplementary figures [file mmc1.pdf]

Supplementary Figure 1

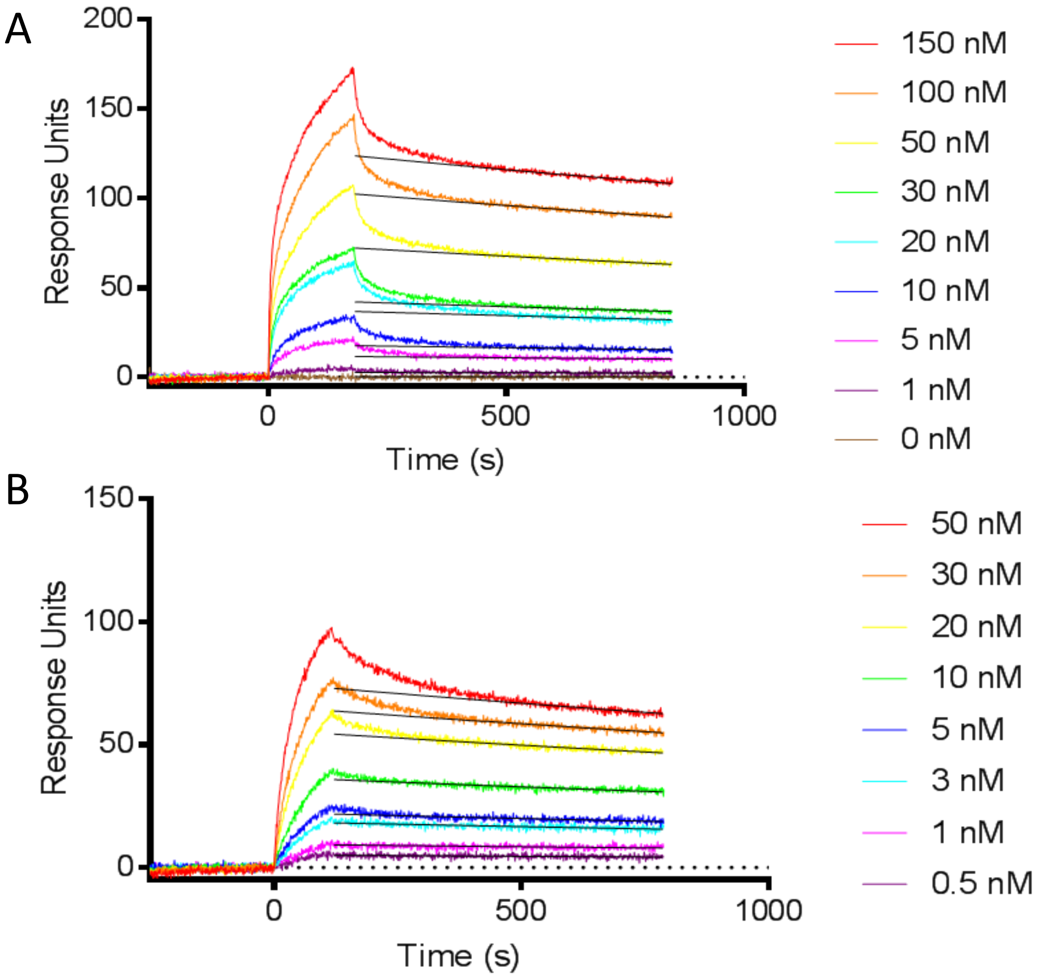

Supplementary figure 1: A) BMPER titrations onto a GLC chip with Tsg immobilised with fitting of the k_{off} rates to BMPER, overlaid in black. B) N-BMPER titrations onto a GLC chip with Tsg immobilised with fitting of the k_{off} rates to N-BMPER, overlaid in black.

Supplementary Figure 2

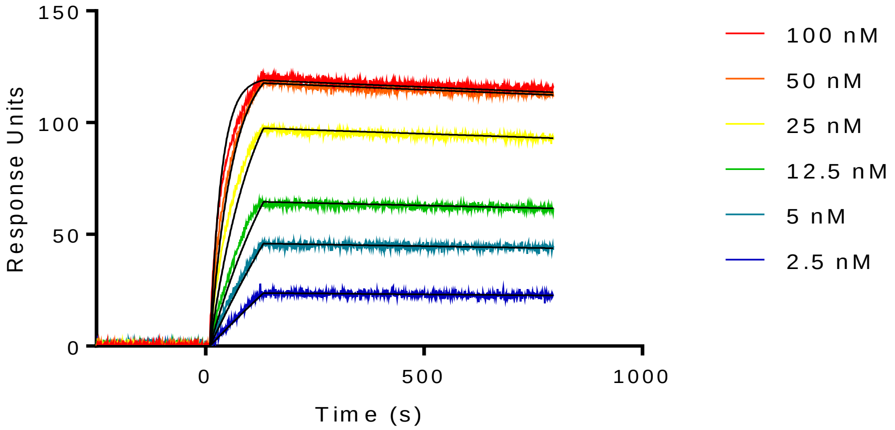

Supplementary Figure 2. N-BMPER binding Chordin vWC2-3.
N-BMPER titrations onto a SPR GLC (Bio-Rad) chip with chordin vWFC2-3 immobilised (200 RU) with real time binding curves, fitted with a 1:1 Langmuir binding model, overlaid in black.

Supplementary Figure 3

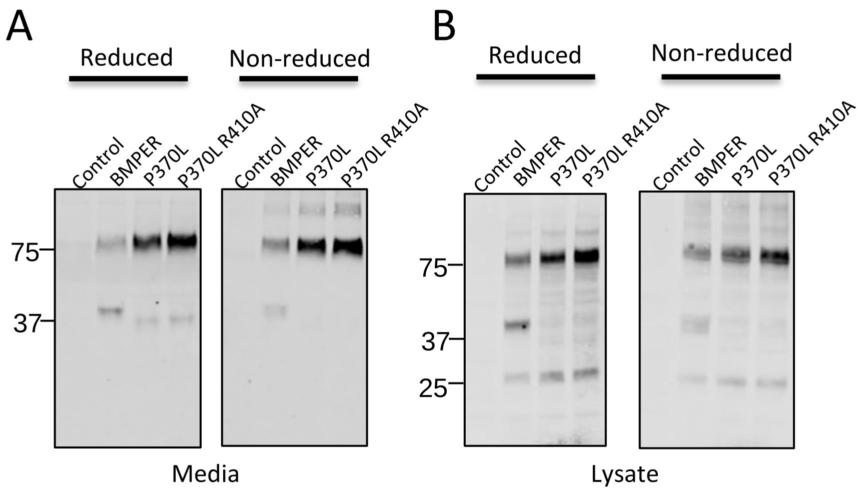

Supplementary figure 3: The conditioned media and cell lysates of BMPER and BMPER double mutant P370LR410A cell-lines and non-transduced control cells were probed by Western blot using an anti-V5 antibody. Reduced and non-reduced conditioned media (A) and cell lysates (B) from transfected HEK293EBNA cells were analysed.

Supplementary Figure 4

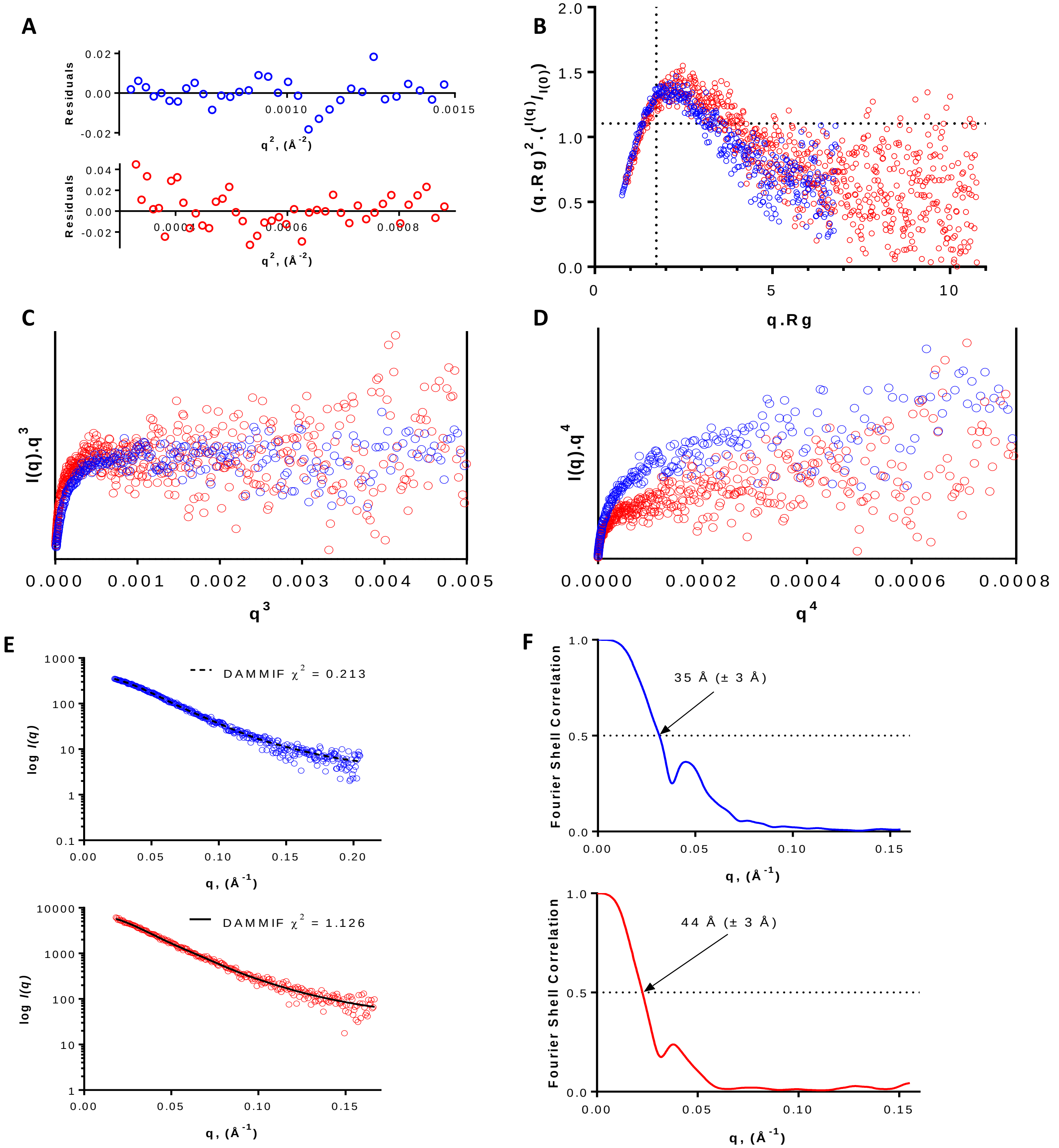

Supplementary Figure 4. A) Residual plots corresponding to the Guinier plots for N-BMPER (blue) and FL-BMPER (red) in Figure 5B. B) Normalised Kratky plot showing that N-BMPER (blue) and FL-BMPER (red) are folded, elongated molecules. The globularity point is indicated by the cross-hairs with N-BMPER and FL-BMPER displaying peak maxima to the right of this point indicating that they are elongated and not globular. C) SIBYLS and (D) Porod-Debye plots showing that both N-BMPER (blue) and FL-BMPER (red) have flexibility and are not rigid systems. E) Scatter showing the fit of the DAMMIF models to each of N-BMPER (blue) and FL-BMPER (red). F) FSC curves for N-BMPER (blue) and FL-BMPER (red) with an FSC of 0.5 showing resolutions of 44 (± 3) Å and 35 (± 3) Å, with bead variabilities of 38.0 and 28.2 respectively.

Supplementary Figure 5

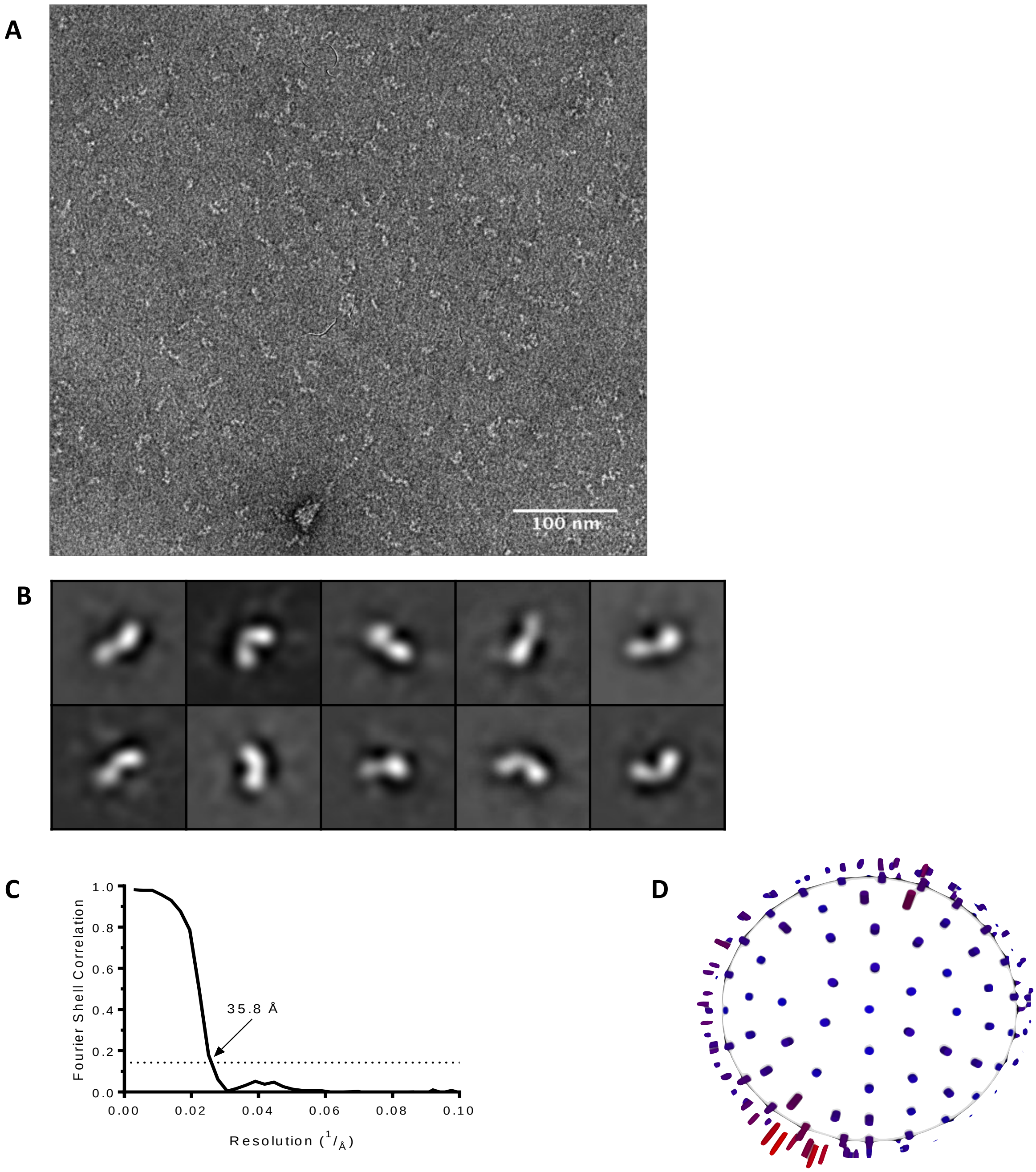

Supplementary Figure 5. A) Negatively stained transmission EM image for FL-BMPER at 30,000 times magnification (Scale bar, 100 nm). B) Selection of reference-free class averages of BMPER. Particles selected using a 25.8 nm box size. C) Fourier shell correlation plotted as a function of resolution. The final resolution was ascertained using a 0.143 cut off. D) Euler angles of the 3D reconstruction of BMPER show a high distribution of particles.
